# Rosuvastatin improves endothelial function in patients with inflammatory joint diseases, longitudinal associations with atherosclerosis and arteriosclerosis: results from the RORA-AS statin intervention study

**DOI:** 10.1186/s13075-015-0795-y

**Published:** 2015-10-08

**Authors:** Eirik Ikdahl, Jonny Hisdal, Silvia Rollefstad, Inge C. Olsen, Tore K. Kvien, Terje R. Pedersen, Anne Grete Semb

**Affiliations:** Preventive Cardio-Rheuma Clinic, Department of Rheumatology, Diakonhjemmet Hospital, P.O. Box 23, Vinderen, 0319 Oslo, Norway; Section of Vascular Investigations, Oslo University Hospital Aker, P.O. Box 0424, Blindern, 0316 Oslo, Norway; Department of Rheumatology, Diakonhjemmet Hospital, P.O. Box 23, Vinderen, 0319 Oslo, Norway; Centre of Preventive Medicine, Oslo University Hospital, Kirkeveien 166, 0450, Oslo, Norway; Faculty of Medicine, University of Oslo, P.O. Box 1078, Blindern, 0316 Oslo, Norway

## Abstract

**Introduction:**

Endothelial dysfunction is an early step in the atherosclerotic process and can be quantified by flow-mediated vasodilation (FMD). Our aim was to investigate the effect of long-term rosuvastatin therapy on endothelial function in patients with inflammatory joint diseases (IJD) with established atherosclerosis. Furthermore, to evaluate correlations between change in FMD (ΔFMD) and change in carotid plaque (CP) height, arterial stiffness [aortic pulse wave velocity (aPWV) and augmentation index (AIx)], lipids, disease activity and inflammation.

**Methods:**

Eighty-five statin-naïve patients with IJD and ultrasound-verified CP (rheumatoid arthritis: n = 53, ankylosing spondylitis: n = 24, psoriatic arthritis: n = 8) received rosuvastatin treatment for 18 months. Paired-samples *t* tests were used to assess ΔFMD from baseline to study end. Linear regression models were applied to evaluate correlations between ∆FMD and cardiovascular risk factors, rheumatic disease variables and medication.

**Results:**

The mean ± SD FMD was significantly improved from 7.10 ± 3.14 % at baseline to 8.70 ± 2.98 % at study end (*p* < 0.001). Improvement in AIx (*p* < 0.05) and CP height reduction (*p* = 0.001) were significantly associated with ΔFMD (dependent variable).

**Conclusions:**

Long-term lipid lowering with rosuvastatin improved endothelial function in IJD patients with established atherosclerotic disease. Reduced arterial stiffness and CP regression were longitudinally correlated with the improvement in endothelial function measured by FMD.

**Trial registration:**

ClinicalTrials.gov NCT01389388. Registered 16 April 2010.

**Electronic supplementary material:**

The online version of this article (doi:10.1186/s13075-015-0795-y) contains supplementary material, which is available to authorized users.

## Introduction

Patients with inflammatory joint diseases (IJD), including rheumatoid arthritis (RA), ankylosing spondylitis (AS) and psoriatic arthritis (PsA) have reduced endothelial function compared to the general population [1]. According to hypotheses regarding development of atherosclerosis, the progressive impairment of endothelial function is an early step in the developing process of atherosclerotic plaques [2]. Furthermore, restoration of endothelial function has been proposed as an important mediator of atherosclerotic regression [3].

Flow-mediated vasodilation (FMD) of the brachial artery is a widely used noninvasive technique that quantifies endothelial function [4]. Reduced FMD has been shown to predict cardiovascular disease (CVD) events in other high-risk populations and in the general population [5]. Moreover, decreasing FMD correlates with the presence of carotid plaques (CP) [6], which are highly prevalent in RA patients [7]. The European recommendations on CVD prevention classify CP as a very high CVD risk factor [8]. Due to the ethical implications of not giving statin therapy to patients with CP, the ROsuvastatin in Rheumatoid Arthritis, Ankylosing Spondylitis and other inflammatory joint diseases (RORA-AS) study was a single-arm statin study from which we have previously reported that rosuvastatin therapy induced CP height regression in IJD patients [9].

The main objective of the present secondary endpoint analyses from the RORA-AS study was to evaluate the effect of long-term lipid lowering with rosuvastatin on FMD in IJD patients with established atherosclerotic disease. Furthermore, we aimed to examine if the change in FMD (∆FMD) induced by rosuvastatin would be correlated with CP height reduction. In addition, we evaluated if ∆FMD from baseline to study end could be predicted by, or was correlated with, the baseline levels of or the longitudinal changes in arterial stiffness, lipids, inflammatory parameters, rheumatic disease activity, carotid intima-media thickness (c-IMT) or rosuvastatin dose.

## Methods

The study design of the open, prospective RORA-AS study has previously been described in detail [9]. In short, IJD patients with ultrasound-verified CP were treated with rosuvastatin for 18 months. The initial rosuvastatin dose (20 mg once daily (o.d.), except for patients > 70 years who received 5 mg) was doubled every fortnight, until low-density lipoprotein (LDL-c) target (<1.8 mmol/L) or maximal rosuvastatin dose (40 mg o.d.) was reached. The LDL-c target was defined in accordance with the most recent European guidelines [8]. The feasibility of reaching this target in IJD patients with low rates of adverse events has previously been reported [9, 10]. All patients signed an informed consent and the study was approved by the Norwegian South East Regional Health committee and registered with ClinicalTrials.gov Id: NCT01389388. The European Union Drug Regulating Authorities Clinical Trials (EudraCT) number is 2008-005551-20.

Endothelial function was assessed by the FMD method [4]. As the FMD measurement is dynamic in nature and can be impacted by several factors, all recordings were performed in fasting patients, including no smoking or medication. In addition, all RORA-AS study visits were independent from other visits to the rheumatology outpatient clinic throughout the study. Accordingly, there existed no systematic tendency toward the administration of disease-modifying antirheumatic drugs (DMARDs) in the days prior to the FMD measurements that would influence the results. All recordings were done with the patient in a supine position using a Vivid 7 scanner (GE Vingmed Ultrasound, Horten, Norway) with a 10 MHz transducer that was placed in a fixed position and stabilized with a custom-made tripod to optimize the stability. The results were analyzed offline from jpg images extracted from video files using Brachial Analyzer software (Medical Imaging Applications LLC, Coralville, IA, USA). FMD percentage was calculated as the percentage change between the baseline diameter and the largest diameter of the brachial artery within the first 2 minutes after 5 minutes occlusion of the right forearm. The examiner had no information pertaining to further patient characteristics. Recordings were performed and analyses were supervised by the same experienced ultrasonographer (JH) in accordance with international guidelines [4], and the intra- and inter-reader reliability for FMD showed a good correlation (intraclass correlations = 0.96 for both). Flow of the brachial artery was not obtained during the FMD examinations. The mean diameter of the brachial artery for all patients at both baseline and 18 months was plotted against time from 0 to 120 seconds and the area under the curve (AUC_0–120_) for both graphs were calculated as a millimeters multiplied with seconds (mm × sec) value.

To assess arterial stiffness, augmentation index (AIx) and aortic pulse wave velocity (aPWV) were measured by the Sphygmocor apparatus (Atcor Medical Pty Ltd., West Ryde, Australia) according to guidelines and as previously described [11].

### Statistics

Descriptive statistics are expressed as number (%) for dichotomized variables, and mean ± standard deviation (SD) and median and interquartile range (IQR) for normally and non-normally distributed variables, respectively. Diagnosis groups were compared using analysis of variance (ANOVA), and chi-square tests as appropriate, in addition to the nonparametric Kruskal-Wallis test for number of CP. Non-normally distributed variables were logarithmically transformed.

The change in FMD from baseline to study end was analyzed using a paired-samples *t* test.

Both adjusted and unadjusted linear regression analyses with ∆FMD as the dependent variable were applied to:

1. Evaluate if ∆FMD could be predicted by baseline demographic data (age, gender), use of biologic DMARDs (bDMARDs), inflammation [C-reactive protein (CRP), erythrocyte sedimentation rate (ESR)], rheumatic disease activity [disease activity score in 28 joints (DAS28), ankylosing spondylitis disease activity score (ASDAS) or lipids [(low-density lipoprotein (LDL-c), high-density lipoprotein (HDL-c)]
2. Explore if ∆FMD was correlated with the changes in CP height, inflammation, rheumatic disease activity, lipids, arterial stiffness, c-IMT and rosuvastatin dose from baseline to study end

Model assumptions and validity were assessed using residuals. Two-sided *p* values <0.05 were considered significant. Statistical analyses were performed using the Statistical Package for the Social Sciences version 21 (IBM SPSS Statistics for Windows, Armonk, NY, USA).

## Results

The RA (n = 53), AS (n = 24) and PsA (n = 8) patients were comparable concerning baseline characteristics (Table 1). However, we observed expected differences concerning gender (*p* = 0.01), baseline HDL-c levels (*p* = 0.02) and use of synthetic disease-modifying antirheumatic drugs (sDMARDS) (*p* = 0.002), attributable to the distinct characteristics of the three disease entities. Changes in lipid levels and CP height in the RORA-AS study has previously been reported [9]. An additional table has been provided that shows these changes in detail (see Additional file 1).

**Table 1:** Patient characteristics

	RA	AS	PsA	All	*p* value
					RA/AS/PsA
Number, n (%)	53	24	8	85	
Age, median (IQR)	61.0 (56.0–68.0)	59.5 (53.5–64.0)	62.0 (56.3–66.5)	61.0 (56.0–67.0)	0.26
Sex female/male, n (%)	40/13 (75.5/24.5)	8/16 (33.3/66.7)	3/5 (37.5/62.5)	51/34 (60.0/40.0)	0.001
Disease duration, median (IQR)	16.0 (8.0–22.8)	23.5 (11.5–30.0)	14.0 (4.0–30.5)	18.0 (8.3–26.0)	0.29
DAS28, mean ± SD	2.51 ± 0.88	–	–	–	–
ASDAS, mean ± SD	–	5.96 ± 2.95	–	–	–
CVD risk factors					
Smoke, n (%)	10 (18.9)	3 (12.5)	1 (12.5)	14 (16.5)	0.75
BMI, mean ± SD	25.1 ± 3.3	25.2 ± 2.6	25.9 ± 2.9	25.2 ± 3.1	0.81
TC (mmol/L), mean ± SD	6.41 ± 1.20	6.25 ± 0.87	6.24 ± 1.17	6.35 ± 1.11	0.81
HDL-c (mmol/L), mean ± SD	1.83 ± 0.53	1.53 ± 0.43	1.45 ± 0.31	1.71 ± 0.50	0.02
TG (mmol/L), median (IQR)	1.21 (0.89–1.57)	1.44 (1.02–1.99)	1.13 (0.78–2.70)	1.22 (0.89–1.75)	0.40
LDL-c (mmol/L), mean ± SD	3.98 ± 1.09	4.03 ± 0.85	4.10 ± 0.97	4.01 ± 1.01	0.95
sBP (mmHg), mean ± SD	142.8 ± 21.7	144.8 ± 14.3	151.1 ± 25.3	144.1 ± 20.2	0.55
dBP (mmHg), mean ± SD	83.2 ± 9.2	84.6 ± 8.5	88.5 ± 11.5	84.1 ± 9.3	0.30
Comorbidities, n (%)					
HT	32 (60.4)	17 (70.8)	6 (75.0)	55 (64.7)	0.55
Diabetes	4 (7.5)	2 (8.3)	1 (12.5)	7 (8.2)	0.84
CVD	5 (9.4)	4 (16.7)	0 (0)	9 (10.6)	0.38
CP, median (range)	1.0 (1.0–2.0)	1.0 (1.0–3.0)	2.5 (2.0–3.0)	1.0 (1.0–2.0)	0.09
Biomarkers, mean ± SD					
ESR (mm/h)	15.1 ± 10.3	16.2 ± 10.5	12.4 ± 9.8	15.9 ± 10.1	0.32
White blood cells (10^9^/L)	7.38 ± 2.97	7.74 ± 3.44	6.68 ± 1.72	6.67 ± 1.37	0.54
CRP (mg/L), median (IQR)	3.00 (1.00–4.00)	1.00 (1.00–3.00)	4.00 (2.00–7.25)	2.00 (1.00–4.00)	0.16
Hb (g/100 mL)	14.1 ± 1.1	14.0 ± 1.0	14.4 ± 1.0	14.0 ± 1.2	0.25
Creatinine (μmol/L)	72.0 ± 13.4	70.9 ± 13.7	74.6 ± 13.9	70.8 ± 8.3	0.52
AST (U/L)	28.1 ± 10.1	28.2 ± 11.2	28.8 ± 9.5	24.9 ± 3.6	0.64
ALT (U/L)	29.8 ± 19.5	28.6 ± 19.6	32.4 ± 22.0	29.6 ± 8.6	0.74
CK (U/L)	78.2 ± 40.0	99.0 ± 48.1	66.4 ± 28.7	82.9 ± 42.5	0.08
Medication, n (%)					
Prednisolone	17 (32.1)	2 (8.3)	2 (25.0)	21 (24.7)	0.08
NSAIDs	20 (37.7)	12 (50.0)	3 (37.5)	35 (41.2)	0.58
sDMARDs	32 (60.4)	6 (25.0)	7 (87.5)	45 (52.9)	0.002
bDMARDs	17 (32.1)	8 (33.3)	4 (50.0)	29 (34.1)	0.73
a-HT medication	16 (30.2)	4 (16.7)	2 (25.0)	22 (25.9)	0.45

*RA* rheumatoid arthritis, *AS* ankylosing spondylitis, *PsA* psoriatic arthritis, *IQR* interquartile range, *DAS28* disease activity score in 28 joints, *SD* standard deviation, *ASDAS* ankylosing spondylitis disease activity score, *CVD* cardiovascular disease, *BMI* body mass index, *TC* total cholesterol, *HDL-c* high-density lipoprotein cholesterol, *TG* triglycerides, *LDL-c* low-density lipoprotein cholesterol, *sBP* systolic blood pressure, *dBP* diastolic blood pressure, *HT* hypertension (self-reported hypertension, blood pressure >140/90 mmHg or current use of antihypertensive medication), *CP* carotid plaque, *ESR* erythrocyte sedimentation rate, *CRP* C-reactive protein, *Hb* hemoglobin, *AST* aspartate aminotransferase, *ALT* alanine aminotransferase, *CK* creatine kinase, *NSAIDs* nonsteroidal anti-inflammatory drugs, *DMARDs* disease-modifying antirheumatic drug, *sDMARDs* synthetic DMARDs, *bDMARDs* biologic DMARDs, *a-HT* antihypertensive

**Fig. 1:**
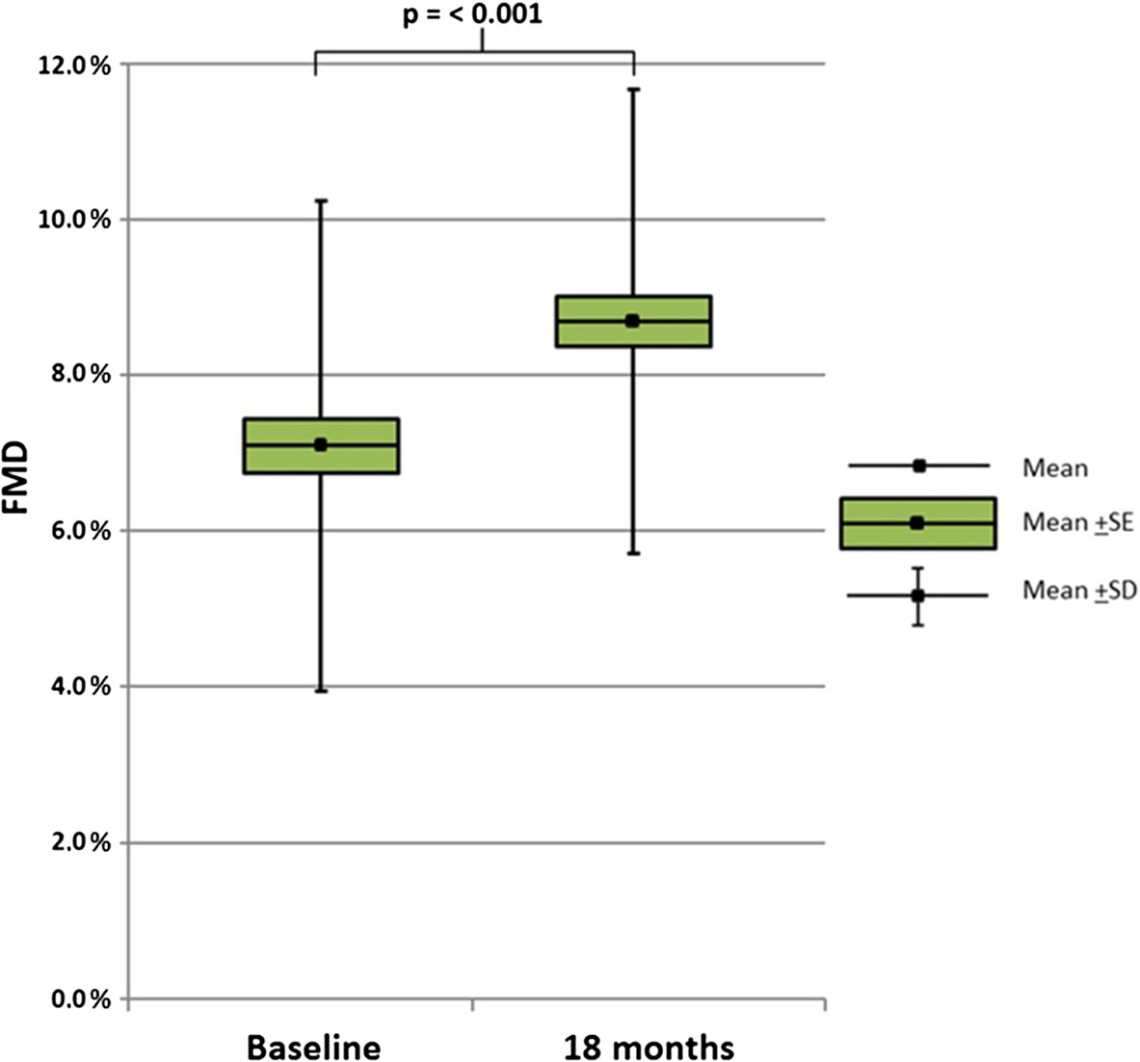
Change in flow-mediated vasodilation (FMD) from baseline to study end. Change in flow-mediated vasodilation (FMD) (%) from baseline to study end in patients with inflammatory joint diseases and established atherosclerosis (n = 85) after 18 months of rosuvastatin treatment. *FMD* flow-mediated vasodilation, *SE* standard error, *SD* standard deviation

**Fig. 2:**
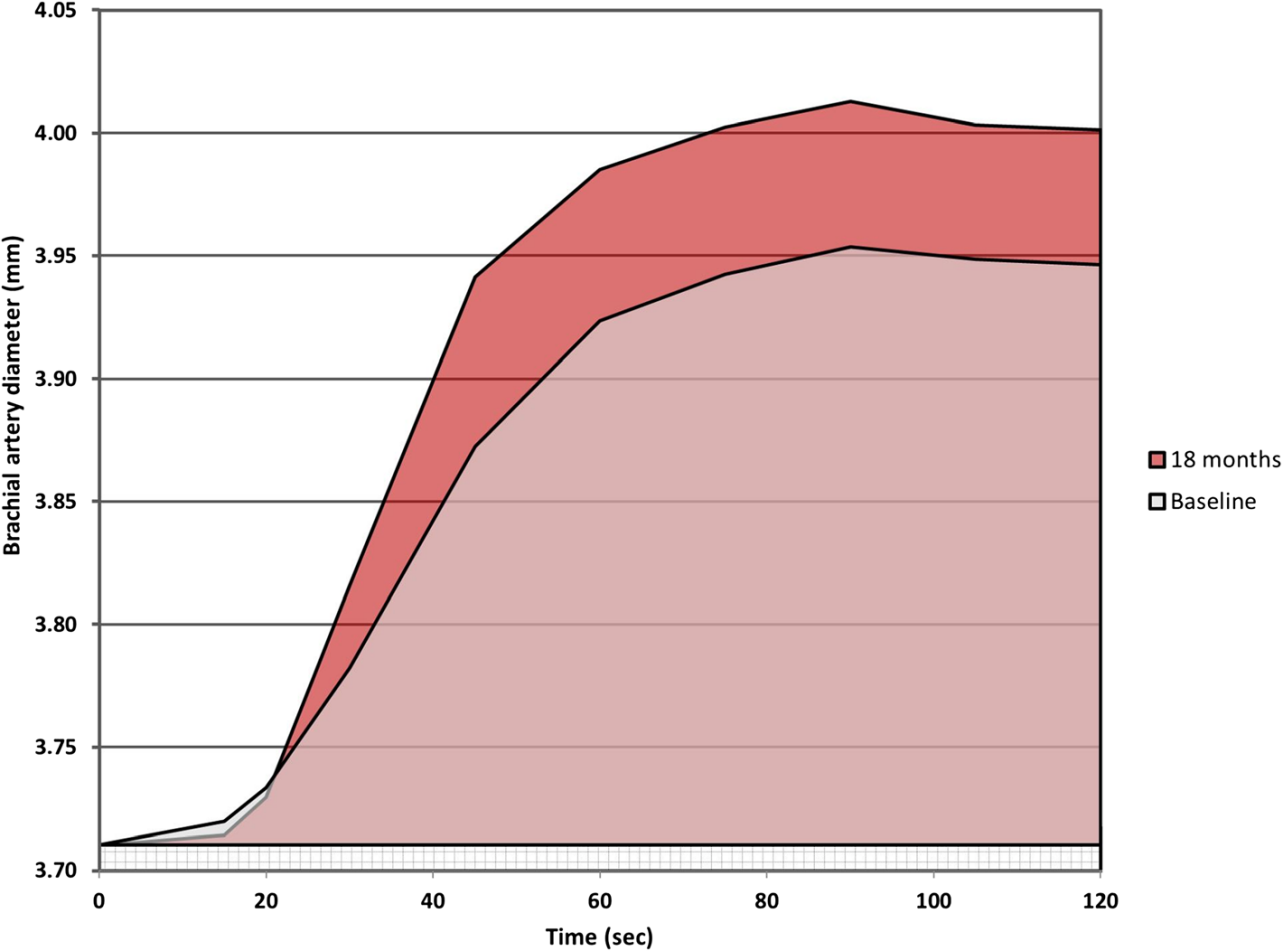
Mean brachial artery diameter plotted against time at baseline and study end. Flow-mediated vasodilation (FMD) mean brachial artery diameter (millimeters) plotted against time (seconds) in patients with inflammatory joint diseases and established atherosclerosis (n = 85), before and after 18 months of rosuvastatin treatment

FMD was improved by 1.6 % from baseline to study end (Fig. 1), reflecting a mean ± SD FMD of 7.10 ± 3.14 % and 8.70 ± 2.98 %, respectively (*p* < 0.001). Figure 2 shows the relative change in vascular diameter as measured by FMD from 0 to 120 seconds. The AUC_0–120_ of the baseline measurements was increased by 27.7 % from baseline (19.3 mm × sec) to study end (24.6 mm × sec).

The adjusted and unadjusted analyses did not reveal any statistically significant predictors of ∆FMD in our study (Table 2). However, in the adjusted linear regression analyses, the changes in AIx and CP height were associated with ∆FMD, in the sense that patients who experienced reduced arterial stiffness measured by AIx and CP height reduction also had increased FMD (*p* < 0.05 and *p* = 0.001, respectively). The estimates from the corresponding unadjusted linear regression analyses were comparable. ∆FMD did not significantly correlate with baseline levels or change in inflammatory biomarkers, rheumatic disease activity, lipids, c-IMT or rosuvastatin dose in the linear regression analyses.

**Table 2:** Unadjusted and adjusted linear regression analyses with flow-mediated vasodilation (ΔFMD) as the dependent variable

		Unadjusted analyses		Adjusted analyses^a^	
		β (95 % CI)	*p* value	β (95 % CI)	*p* value
Age		−0.07 (−0.14, 0.01)	0.09	–	
Gender		0.38 (−0.89, 1.66)	0.55	–	
bDMARDs		−1.07 (−2.31, 0.18)	0.09	–	
CRP	Baseline	−0.01 (−0.18, 0.16)	0.92	−0.02 (−0.18, 0.15)	0.86
	Change	0.04 (−0.01, 0.09)	0.13	0.05 (−0.01, 0.10)	0.08
ESR	Baseline	−0.01 (−0.07, 0.05)	0.83	0.01 (−0.06, 0.08)	0.76
	Change	0.04 (−0.02, 0.09)	0.18	0.04 (−0.01, 0.09)	0.15
DAS28	Baseline	0.24 (−0.82, 1.29)	0.66	0.29 (−0.73, 1.31)	0.57
	Change	0.02 (−1.23, 1.27)	0.98	−0.25 (−1.43, 0.93)	0.67
ASDAS	Baseline	−0.01 (−0.40, 0.38)	0.96	0.08 (−0.53, 0.68)	0.78
	Change	0.14 (−0.31, 0.58)	0.52	0.14 (−0.52, 0.81)	0.63
LDL-c	Baseline	0.01 (0.62, 0.63)	0.98	−0.13 (−0.75, 0.50)	0.69
	Change	−0.17 (−0.91, 0.57)	0.65	−0.06 (−0.80, 0.68)	0.87
HDL-c	Baseline	−0.54 (−1.78, 0.70)	0.39	−0.26 (−1.70, 1.17)	0.72
	Change	−1.33 (−3.18, 0.52)	0.16	−1.43 (−3.28, 0.42)	0.13
AIx, change		−0.09 (−0.18, 0.01)	0.06	−0.09 (−0.18, 0.00)	< 0.05
aPWV, change		−0.07 (−0.58, 0.43)	0.78	−0.14 (−0.66, 0.38)	0.59
CP height, change		−3.51 (−5.28, −1.73)	< 0.001	−3.10 (−4.95, −1.25)	0.001
c-IMT, change		3.55 (−4.51, 11.62)	0.38	3.96 (−4.77, 12.70)	0.37
Rosuvastatin dose		0.02 (−0.03, 0.07)	0.48	0.01 (−0.04, 0.06)	0.76

*bDMARDs* biologic disease-modifying antirheumatic drugs, *CRP* C-reactive protein, *ESR* erythrocyte sedimentation rate, *DAS28* disease activity score in 28 joints, *ASDAS* ankylosing spondylitis disease activity score, *LDL-c* low-density lipoprotein cholesterol, *HDL-c* high-density lipoprotein cholesterol, *AIx* augmentation index, *aPWV* aortic pulse wave velocity, *CP* carotid plaque, *c-IMT* carotid intima-media thickness

^a^Adjusted for age, gender and use of bDMARDs

## Discussion

In the present study, we have shown that endothelial function was significantly improved in IJD patients with established atherosclerosis who received rosuvastatin therapy for 18 months. Furthermore, we have demonstrated that the amelioration of FMD was correlated with CP regression and reduced arterial stiffness.

The improvement in FMD after statin treatment observed in our study is supported by the positive effect by statins on FMD reported from previous studies with smaller IJD patient cohorts and shorter follow-up time [12–14]. Interestingly, a meta-analysis by Inaba et al. reported that 1 % lower FMD was associated with a 13 % increase in risk of future CVD events. Accordingly, the 1.6 % improvement in FMD observed in our study may reflect a highly beneficial clinical effect by rosuvastatin [15].

The negative influence of endothelial dysfunction on the progression of atherosclerosis has previously been reported [16]. We show for the first time that carotid atherosclerosis regression was associated with improvement of FMD in statin-treated patients. Thus, our results support the existing evidence that restoration of endothelial function may be essential in the reversal of atherosclerosis [3]; however, studies to elucidate further details in this pathological process are warranted.

AIx and aPWV are predictors of CVD in the general population [17, 18] and are reported to be higher in patients with IJD [1]. The inverse relation between FMD and arterial stiffness in IJD patients has previously been described [19]. To our knowledge, this is the first report on longitudinal correlation between improved endothelial function and decreasing arterial stiffness. Although endothelial dysfunction and arterial stiffness represent different aspects of vascular disease, the pathogeneses of these vascular biomarkers are closely related [20]. Our results indicate that these biomarkers are also related in the process of atherosclerotic regression.

We found no significant correlation between ∆FMD and the levels of inflammatory biomarkers or rheumatic disease activity in our patients. This may be due to that the patients were largely well treated at baseline and throughout the study.

The lack of a placebo group in the RORA-AS study represents a possible limitation. However, as aging is related to progressive impairment of endothelial function [21], it is unlikely that the improved FMD in our study was a result of natural progression. Data concerning flow of the brachial artery in relation to the FMD measurements would have been preferable; however, such data were not obtained.

## Conclusions

In conclusion, endothelial function was significantly improved in IJD patients with established atherosclerosis receiving long-term lipid-lowering therapy with rosuvastatin. The amelioration of FMD was correlated with reduced arterial stiffness and decreasing CP height and support the hypothesis that restoration of endothelial function is closely related to atherosclerotic regression.

## Additional file

Additional file 1:
**Changes in lipid levels and carotid plaque height in the RORA-AS study.** Description of data: Additional file 1 shows carotid plaque height, carotid intima-media thickness, LDL cholesterol, HDL cholesterol, triglycerides and total cholesterol at baseline and at study end. It also shows the mean change and corresponding *p* values for these parameters during the course of the study. (PDF 185 kb)

## Abbreviations

AIx: augmentation index
ANOVA: analysis of variance
aPWV: aortic pulse wave velocity
AS: ankylosing spondylitis
ASDAS: ankylosing spondylitis disease activity score
AUC: area under the curve
bDMARDs: biologic disease modifying antirheumatic drugs
c-IMT: carotid intima-media thickness
CP: carotid plaques
CRP: C-reactive protein
CVD: cardiovascular disease
DAS28: disease activity score in 28 joints
ESR: erythrocyte sedimentation rate
FMD: flow-mediated vasodilation
HDL-c: high-density lipoprotein
IJD: inflammatory joint diseases
IQR: interquartile range
LDL-c: low-density lipoprotein
PsA: psoriatic arthritis
RA: rheumatoid arthritis
SD: standard deviation
sDMARDs: synthetic disease-modifying antirheumatic drugs

## References

[1] Soltesz P, Kerekes G, Der H, Szucs G, Szanto S, Kiss E (2011). Comparative assessment of vascular function in autoimmune rheumatic diseases: considerations of prevention and treatment. Autoimmun Rev.

[2] Ross R (1999). Atherosclerosis--an inflammatory disease. N Eng J Med.

[3] Francis AA, Pierce GN (2011). An integrated approach for the mechanisms responsible for atherosclerotic plaque regression. Exp Clin Cardiol.

[4] Corretti MC, Anderson TJ, Benjamin EJ, Celermajer D, Charbonneau F, Creager MA (2002). Guidelines for the ultrasound assessment of endothelial-dependent flow-mediated vasodilation of the brachial artery: a report of the International Brachial Artery Reactivity Task Force. J Am Coll Cardiol.

[5] Ras RT, Streppel MT, Draijer R, Zock PL (2013). Flow-mediated dilation and cardiovascular risk prediction: a systematic review with meta-analysis. Int J Cardiol.

[6] Rundek T, Hundle R, Ratchford E, Ramas R, Sciacca R, Di Tullio MR (2006). Endothelial dysfunction is associated with carotid plaque: a cross-sectional study from the population based Northern Manhattan Study. BMC Cardiovasc Disord.

[7] Semb AG, Rollefstad S, Provan SA, Kvien TK, Stranden E, Olsen IC (2013). Carotid plaque characteristics and disease activity in rheumatoid arthritis. J Rheumatol.

[8] Perk J, De Backer G, Gohlke H, Graham I, Reiner Z, Verschuren WM (2012). European Guidelines on cardiovascular disease prevention in clinical practice (version 2012): the Fifth Joint Task Force of the European Society of Cardiology and Other Societies on Cardiovascular Disease Prevention in Clinical Practice (constituted by representatives of nine societies and by invited experts). Atherosclerosis.

[9] Rollefstad S, Ikdahl E, Hisdal J, Olsen IC, Holme I, Hammer HB (2015). Rosuvastatin induced carotid plaque regression in patients with inflammatory joint diseases: the RORA-AS study. Arthritis Rheumatol.

[10] Rollefstad S, Kvien TK, Holme I, Eirheim AS, Pedersen TR, Semb AG (2013). Treatment to lipid targets in patients with inflammatory joint diseases in a preventive cardio-rheuma clinic. Ann Rheum Dis.

[11] Provan SA, Semb AG, Hisdal J, Stranden E, Agewall S, Dagfinrud H (2011). Remission is the goal for cardiovascular risk management in patients with rheumatoid arthritis: a cross-sectional comparative study. Ann Rheum Dis.

[12] Maki-Petaja KM, Booth AD, Hall FC, Wallace SM, Brown J, McEniery CM (2007). Ezetimibe and simvastatin reduce inflammation, disease activity, and aortic stiffness and improve endothelial function in rheumatoid arthritis. J Am Coll Cardiol.

[13] El-Barbary AM, Hussein MS, Rageh EM, Hamouda HE, Wagih AA, Ismail RG (2011). Effect of atorvastatin on inflammation and modification of vascular risk factors in rheumatoid arthritis. J Rheumatol.

[14] Hermann F, Forster A, Chenevard R, Enseleit F, Hurlimann D, Corti R (2005). Simvastatin improves endothelial function in patients with rheumatoid arthritis. J Am Coll Cardiol.

[15] Inaba Y, Chen JA, Bergmann SR (2010). Prediction of future cardiovascular outcomes by flow-mediated vasodilatation of brachial artery: a meta-analysis. Int J Cardiovasc Imaging.

[16] Gossl M, Yoon MH, Choi BJ, Rihal C, Tilford JM, Reriani M (2014). Accelerated coronary plaque progression and endothelial dysfunction: serial volumetric evaluation by IVUS. JACC Cardiovasc Imaging.

[17] Mattace-Raso FU, van der Cammen TJ, Hofman A, van Popele NM, Bos ML, Schalekamp MA (2006). Arterial stiffness and risk of coronary heart disease and stroke: the Rotterdam Study. Circulation.

[18] Weber T, Auer J, O'Rourke MF, Kvas E, Lassnig E, Berent R (2004). Arterial stiffness, wave reflections, and the risk of coronary artery disease. Circulation.

[19] Soltesz P, Der H, Kerekes G, Szodoray P, Szucs G, Danko K (2009). A comparative study of arterial stiffness, flow-mediated vasodilation of the brachial artery, and the thickness of the carotid artery intima-media in patients with systemic autoimmune diseases. Clin Rheumatol.

[20] Correia ML, Haynes WG (2007). Arterial compliance and endothelial function. Curr Diab Rep.

[21] Seals DR, Jablonski KL, Donato AJ (2011). Aging and vascular endothelial function in humans. Clin Sci (Lond).

