# Supplementary material for: Rosuvastatin improves endothelial function in patients with inflammatory joint diseases, longitudinal associations with atherosclerosis and arteriosclerosis: results from the RORA-AS statin intervention study
Source: Arthritis Res Ther. 2015 Oct 8;17:279. doi: 10.1186/s13075-015-0795-y (PMC4597440; doi:10.1186/s13075-015-0795-y)
Supplement: Additional file 1: — Changes in lipid levels and carotid plaque height in the RORA-AS study. Description of data: Additional file 1 shows carotid plaque height, carotid intima-media thickness, LDL cholesterol, HDL cholesterol, triglycerides and total cholesterol at baseline and at study end. It also shows the mean change and corresponding p values for these parameters during the course of the study. (PDF 185 kb) [file 13075_2015_795_MOESM1_ESM.pdf]

Additional file 1. Changes in lipid levels and carotid plaque height in the RORA-AS study

	**Baseline Mean±SD**	**18 months Mean±SD**	**Mean difference (95% CI)**	**p-value**
**CP height** (mm)	1.93±0.53	1.73±0.49	-0.20 (-0.28, -0.12)	<0.001
**c-IMT** (mm)	0.72±0.15	0.71±0.14	-0.01 (-0.03, 0.01)	0.21
**LDL-cholesterol** (mmol/L)	4.01±1.01	1.71±0.42	-2.30 (-2.48, -2.11)	<0.001
**HDL-cholesterol** (mmol/L)	1.71±0.50	1.78±0.51	0.07 (0.00, 0.14)	0.05
**Triglycerides** (mmol/L)	1.45±0.83	1.08±0.45	-0.37 (-0.51, -0.22)	<0.001
**Total cholesterol** (mmol/L)	6.35±1.11	3.97±0.64	-2.38 (-2.59, -2.17)	<0.001

SD: Standard deviation of the mean, CI: Confidence interval, CP: Carotid plaque, c-IMT: carotid intima-media thickness, LDL: Low-density lipoprotein, HDL: High-density lipoprotein.
